# Long-Term Oxidation of Zirconium Alloy in Simulated Nuclear Reactor Primary Coolant—Experiments and Modeling

**DOI:** 10.3390/ma16072577

**Published:** 2023-03-24

**Authors:** Iva Betova, Martin Bojinov, Vasil Karastoyanov

**Affiliations:** 1 Institute of Electrochemistry and Energy Systems, Bulgarian Academy of Sciences, 1113 Sofia, Bulgaria; 2 Department of Physical Chemistry, University of Chemical Technology and Metallurgy, 1756 Sofia, Bulgaria

**Keywords:** zirconium alloy, nuclear reactor primary coolant, electrochemical impedance spectroscopy, oxidation model, compressive stress, ionic space charge

## Abstract

Oxidation of Zr-1%Nb fuel cladding alloy in simulated primary coolant of a pressurized water nuclear reactor is followed by in-situ electrochemical impedance spectroscopy. In-depth composition and thickness of the oxide are estimated by ex-situ analytical techniques. A kinetic model of the oxidation process featuring interfacial reactions of metal oxidation and water reduction, as well as electron and ion transport through the oxide governed by diffusion-migration, is parameterized by quantitative comparison to impedance data. The effects of compressive stress on diffusion and ionic space charge on migration of ionic point defects are introduced to rationalize the dependence of transport parameters on thickness (or oxidation time). The influence of ex-situ and in-situ hydrogen charging on kinetic and transport parameters is also studied.

## 1. Introduction

A typical pressurized water reactor (PWR) contains around 200 fuel assemblies, with each fuel assembly featuring hundreds of fuel rods. A fuel rod is composed of fuel pellets stacked inside a cladding tube [1,2], The mechanical integrity of the cladding is essential to nuclear safety because it provides the first barrier for fission products. The technical specifications used to choose PWR cladding material have long been: (1) high neutron transparency, i.e., low thermal neutron cross sections, (2) good thermal conductivity, (3) low creep rate, (4) good ultimate elongation, (5) sound mechanical properties, and (6) good corrosion resistance [1]. 

The trend towards more severe operating conditions for this type of structural material, induced by the need to extend the lifetime and increase fuel burnup, leads to a need for a detailed characterization of correlations between composition, microstructure, and morphology of such alloys, growth kinetics of oxide layers on them, and their susceptibility to localized corrosion [1]. Even if the qualitative picture of the oxidation process of Zr alloys in nuclear power plant coolants is well established, the intimate mechanism of the transfer of matter and charge through the oxide remains largely unclear. It is generally believed that the growth of the oxide proceeds according to the so-called coupled-currents mechanism [3]. According to that mechanism, transport of oxygen by a vacancy mechanism along grain boundaries of the already formed zirconium oxide is the rate-determining step of the overall reaction. It is assumed that water reduction at the oxide/coolant interface consumes electrons generated by metal oxidation, electronic conduction in the oxide proceeding along preferred pathways associated with secondary phase particles incorporated into the zirconium oxide matrix [1,4,5]. The extent of the coupling of the electron and ion fluxes is not quantitatively assessed.

Increasing the concentration of lithium hydroxide in PWRs with higher fuel burnup and fuel cycles longer than 12 months is necessary to maintain the primary coolant pH within an acceptable range. Higher lithium concentrations associated with supercooled condensate boiling can lead to crud formation on fuel cladding and reactor internals. In turn, crud formation induces axial power anomaly (AOA) and localized corrosion [6,7,8,9]. The mechanism of AOA is still unclear, but a number of studies have indicated a correlation between concentration of alkali, boric acid, zirconium alloy composition, and occurrence of this type of anomaly. The use of KOH in water cooled—water moderated energy reactor (WWER) coolants have considerable advantages due to its higher solubility, and accordingly, a weaker tendency towards crud formation on fuel cladding and internals. All this added complexity calls for the use of advanced in-situ methods for the characterization of oxidation processes of Zr alloys. 

A promising non-destructive characterization technique is electrochemical impedance spectroscopy (EIS), which has been used to study zirconium alloys in WWER and PWR coolants for several decades now [10,11,12,13,14,15,16,17,18]. Despite numerous experimental data found in the literature, EIS has been used almost exclusively to assess oxide conductivity as depending on environmental conditions. Only a few deterministic models that describe quantitatively the whole impedance spectrum were advanced on the basis of the Point Defect Model (PDM) [13,14,15] and the Mixed-Conduction Model (MCM) [19,20]. 

The aim of the present paper is to further develop and test a deterministic model of growth of a protective oxide layer on a Zr-1%Nb alloy. The model features electrochemical processes at the alloy/oxide and oxide/coolant interfaces coupled via diffusion-migration of point defects in the oxide at the atomic and mesoscopic level. The model is based on the generalized quantitative model of growth of passive oxide films in high temperature electrolytes, the MCM, which has recently been adapted to interpret initial stages of corrosion of zirconium alloys in PWR and WWER primary coolants [19,20,21]. First, experimental data on the oxidation of the alloy in WWER coolant with or without LiOH are presented. Measurements on samples that were ex-situ and in-situ electrochemically charged with hydrogen are also discussed. The thickness and in-depth elemental composition of oxides is estimated by Glow Discharge Optical Emission Spectrometry (GDOES) and scanning electron microscopy (SEM) of oxide cross sections. Further, a new version of the model that considers the influence of compressive stress generated during growth on oxygen diffusion and space charge on the electric field strength in the oxide is described. This extension allows rationalization of the thickness (i.e., oxidation time) dependences of diffusion and migration parameters. Finally, the relative importance of rate constants, diffusion coefficients, and field strength for the kinetics of oxide growth and hydrogen pick-up is discussed.

## 2. Materials and Methods

Working electrodes were cut from Zr-1%Nb fuel cladding tubes. The chemical composition of the alloy is presented in Table 1. Pretreatment of electrodes consisted of mechanical polishing with emery paper grade 1200, chemical polishing in a mixture of 30% HNO_3_ (70%) + 30% H_2_SO_4_ (96%) + 9% HF (50%) + 31% H_2_O, and drying with hot air. Experiments were carried out using a three-electrode system with the alloy as working electrode, a Pt (99.9%, Goodfellow) sheet symmetrically arranged around it as a counter electrode, and a Pd (99.9%, Goodfellow) pseudo-reference electrode mounted in close proximity to the tube (at a distance of 2 mm). Pd was continuously polarized with a current of −30 µA against an additional Pt electrode in order to approximate the reversible hydrogen electrode (RHE). To simulate defective microstructure resulting from proton irradiation, samples were subjected either to preliminary cathodic polarization with a current density of −10 mA cm^−2^ in a 0.1 M KOH solution for 24 h at room temperature, galvanostatically (current density −1 mA cm^−2^) or potentiostatically (at −2.0 V vs. RHE) in a beginning-of-cycle WWER primary coolant without LiOH (0.283 mmol kg^−1^ K as KOH and 0.13 mol kg^−1^ B as H_3_BO_3_) at 80 °C. As a result of this type of treatment, hydrogen atoms formed during water reduction are introduced into the alloy structure.

Experiments were conducted in a nominal beginning-of-cycle WWER coolant with a composition of 0.283 mmol kg^−1^ (16 ppm) K as KOH, 0.13 mol kg^−1^ (1400 ppm) B as H_3_BO_3_, with and without the addition of 0.042 mmol kg^−1^ (1 ppm) Li (as LiOH). For its preparation, p.a. H_3_BO_3_, LiOH and KOH (Sigma Aldrich, St. Louis, MO, USA) were used. No NH_3_ was added to the solutions in order to minimize the effect of dissolved H_2_ (formed by decomposition of ammonia at high temperature) on oxidation kinetics. The conductivity of the coolant at room temperature was 37 ± 0.5 µS cm^−1^ (without LiOH) and 42 ± 0.5 µS cm^−1^ (with LiOH), whereas its pH was 6.1 ± 0.1 (without LiOH) and 6.2 ± 0.1 (with LiOH).

All experiments were performed in an autoclave made of 316 L stainless steel (Parr, volume 3.75 dm^3^) connected to a laboratory made re-circulation loop. The respective electrolyte from a 20 dm^3^ reservoir was continuously pumped through the autoclave at a flow rate of 5 dm^3^ h^−1^, resulting in a full re-circulation every 1.5 h. Prior to its use for this type of measurement, the autoclave and all tube connections were pre-oxidized for 168 h at 280 °C in a coolant containing 0.21 mmol kg^−1^ (12 ppm) K as KOH, in order to form a stable protective layer on all surfaces.

In a typical experiment, after mounting the electrodes and filling the loop with coolant, the system was heated to 80 °C and purged with N_2_ (99.999%) for 16 h. The residual dissolved oxygen concentration after this procedure was below 0.31 µmol kg^−1^. After reaching this value, the temperature was gradually increased, and the target value of 300 ± 1 °C at a pressure of 8.8 ± 0.01 MPa was reached in 2–2.5 h. Electrochemical impedance measurements were started at that temperature and were carried out for exposures up to 720 h. A 10030 Compactstat (Ivium, Eindhoven, the Netherlands) operating in floating mode and driven by IviumSoft 4.9 software was used. The frequency range of the measurements was from 30 kHz to 1 mHz at an ac amplitude of 50 mV (rms). The linearity of the spectra was checked by measurements using amplitudes between 10 and 50 mV, whereas causality was ensured using a Kramers-Kronig compatibility test using the procedure of Boukamp [22]. During the first 2–3 days of an experiment, the spectra were recorded every 2–3 h, during the next 3–4 days—every 4 h, and subsequently—every 12 or 24 h, depending on the variation of spectra with time (a typical measurement of an impedance spectrum took about 2–2.5 h). All the experiments were at least triplicated to ensure reproducibility. In order to discriminate more clearly between processes by their time constants in the high-frequency domain, 90% of the ohmic resistance of the coolant between the working and reference electrodes was subtracted from the real part of each spectrum. Complex non-linear fitting of impedance data to the transfer function of the proposed model was performed by a custom routine on an Origin Pro platform (Originlab, Northampton, MA, USA). A Levenberg-Marquardt algorithm with statistical weighting of the respective datasets was used.

In-depth elemental profiles of the samples after exposure were obtained by GDOES over an area of 5 mm^2^ with a GDA750 instrument (Spectruma Analytik, Hof, Germany) equipped with a polychromator (focal length 750 mm and grating of 2400 channels/mm). Typical operating parameters were: primary voltage 950 V, current 9 mA and pressure 3 hPa. Calibration was based on certified reference materials chosen to cover the elements present in a wide range of nickel alloys in the relevant concentration ranges. Cross-sectional scanning electron microscopic images of the samples were also taken after exposure to estimate oxide thickness.

## 3. Results

### 3.1. Electrochemical Impedance Spectroscopy

**Figure 1:**
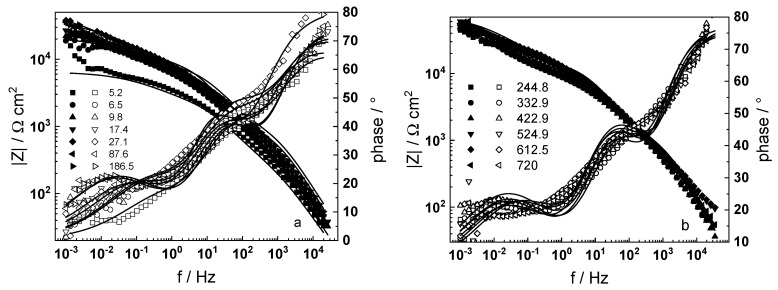
Electrochemical impedance spectra of zirconium alloy in WWER coolant without LiOH at 300 °C depending on the oxidation time. Left ordinate-impedance magnitude (full symbols) and phase shift (open symbols) vs. frequency. Points-experimental data, solid lines-best-fit calculation. The legend gives oxidation time in h.

**Figure 2:**
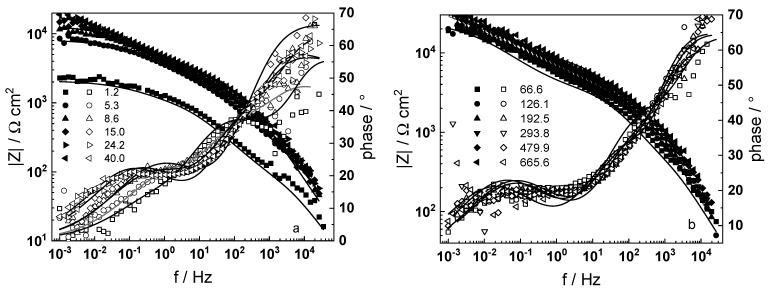
Electrochemical impedance spectra of zirconium alloy in WWER coolant with 0.042 mmol kg^−1^ Li (as LiOH) at 300 °C depending on the oxidation time. Left ordinate-impedance magnitude (full symbols) and phase shift (open symbols) vs. frequency. Points-experimental data, solid lines-best-fit calculation. The legend gives oxidation time in h.

Electrochemical impedance spectra in LiOH-free coolant as depending on exposure time are presented in Figure 1 in Bode coordinates (magnitude and phase shift of impedance vs. frequency). Analogous dependences of the impedance on oxidation time in WWER coolant with the addition of 0.042 mmol kg^−1^ Li (as LiOH) are presented in Figure 2. 

**Figure 3:**
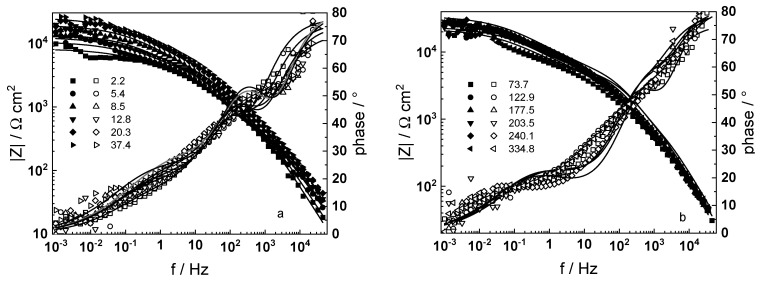
Electrochemical impedance spectra of ex-situ pre-hydrogenated sample in WWER coolant without LiOH at 300 °C depending on the oxidation time. Left ordinate-impedance magnitude (full symbols) and phase shift (open symbols) vs. frequency. Points-experimental data, solid lines-best-fit calculation. The legend gives oxidation time in h.

**Figure 4:**
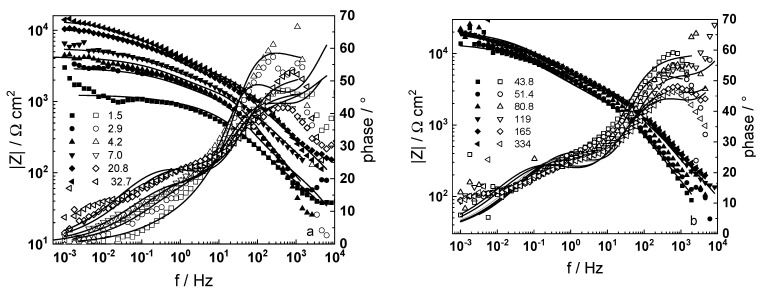
Electrochemical impedance spectra of in-situ pre-hydrogenated sample (galvanostatic mode) in WWER coolant without LiOH at 300 °C depending on the oxidation time. Left ordinate-impedance magnitude (full symbols) and phase shift (open symbols) vs. frequency. Points-experimental data, solid lines-best-fit calculation. The legend gives oxidation time in h.

**Figure 5:**
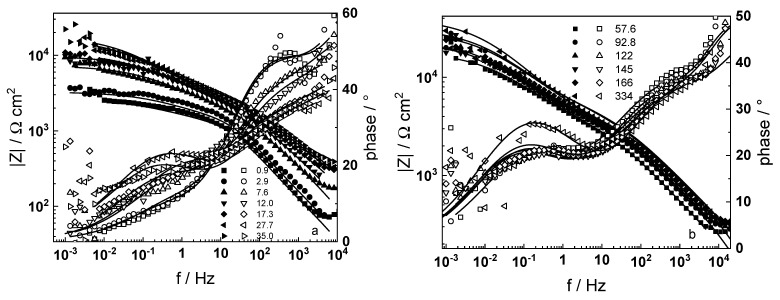
Electrochemical impedance spectra of in-situ pre-hydrogenated sample (potentiostatic mode) in WWER coolant without LiOH at 300 °C depending on the oxidation time. Left ordinate-impedance magnitude (full symbols) and phase shift (open symbols) vs. frequency. Points-experimental data, solid lines-best-fit calculation. The legend gives oxidation time in h.

Impedance spectra of a sample that was pre-hydrogenated for 24 h by polarization with −10 mA cm^−2^ at room temperature in 0.1 M KOH are presented in Figure 3. The respective spectra measured after 24 h cathodic polarization at 80 °C in a WWER coolant without LiOH in galvanostatic (−1 mA cm^−2^) or potentiostatic (−2.0 V vs. RHE) modes are summarized in Figure 4 and Figure 5. 

A common feature of all spectra is the increase of the impedance magnitude at low frequencies (e.g., 1 mHz) with oxidation time, indicating a decrease in the rate of the-limiting step of corrosion. The extent of this decrease depends on oxidation conditions. The larger scatter in the low-frequency data for in-situ hydrogen charged samples is probably due to a dynamic steady state achieved at a larger local variation of transport rates in the oxide. In the phase shift vs. frequency curves, three-time constants are observed, corresponding to the electric properties of the oxide layer, the charge transfer process at the oxide/coolant interface and the ion conduction through the growing film, as discussed also in our previous studies [19,20]. 

### 3.2. Chemical Composition and Thickness

**Figure 6:**
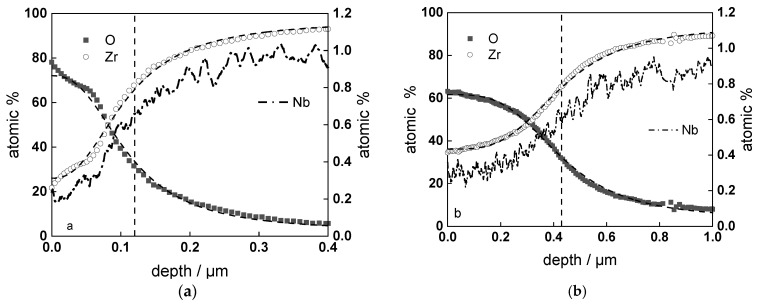
Elemental composition profile of the oxide formed for 24 (**a**) and 720 h (**b**) in WWER coolant without LiOH at 300 °C. Dashed lines show sigmoidal regression of O and Zr profiles to estimate the position of the alloy/oxide interface (shown by vertical line).

**Figure 7:**
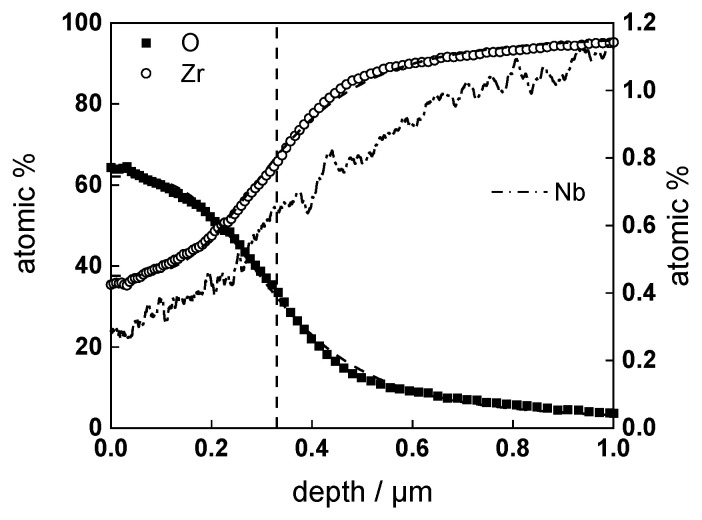
Elemental composition profile of the oxide on Zr-1%Nb alloy formed for 720 h in WWER coolant with LiOH at 300 °C. Dashed lines show sigmoidal regression of O and Zr profiles to estimate the position of the alloy/oxide interface (shown by vertical line).

**Figure 8:**
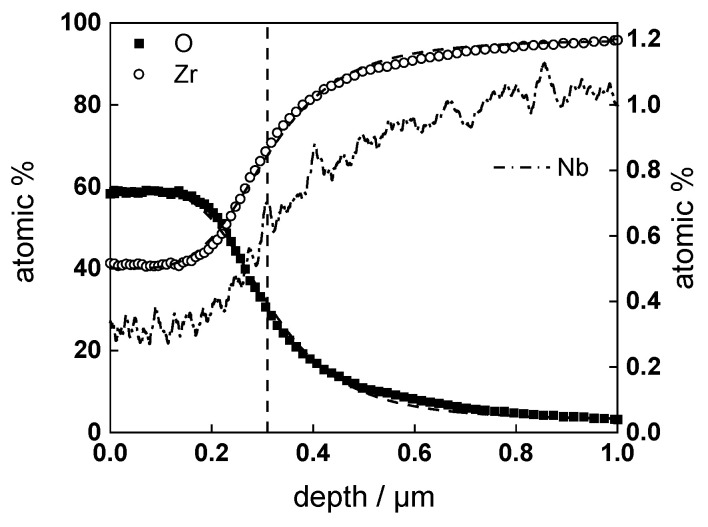
Elemental composition profile of the oxide on formed for 360 h in WWER coolant without LiOH at 300 °C on ex-situ pre-hydrogenated material. Dashed lines show sigmoidal regression of O and Zr profiles to estimate the position of the alloy/oxide interface (shown by vertical line).

GDOES depth profiles of samples after exposure to the experimental conditions described above are presented in Figure 6, Figure 7 and Figure 8. Nb is slightly impoverished in the oxides in comparison to bulk content, and the estimates of the position of alloy/oxide interface using sigmoidal fitting of Zr and O profiles coincide within ±5%. 

**Figure 9:**
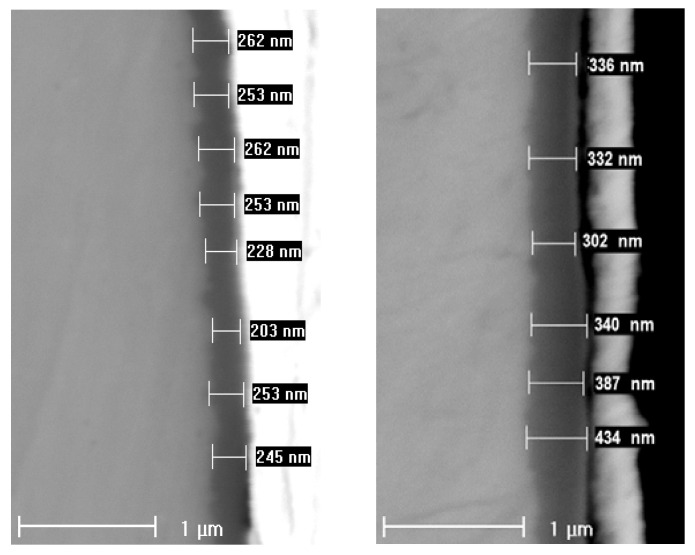
Scanning electron micrographs of the cross-section of oxides formed in WWER coolant without LiOH for 500 (**left**) and 720 h (**right**).

Typical scanning electron micrographs of the cross-sections of samples exposed to WWER coolant without LiOH at 300 °C for 500 and 720 h are shown in Figure 9. It can be concluded that the oxides are uniform and the variation of thickness is less than 5%. No micro-cracks are observed at the magnification used. 

A summary of oxide thicknesses estimated by GDOES and SEM is collected in Table 2 and indicates a good agreement between the two methods. A larger difference was observed for the pre-hydrogenated sample, which could be due to the significant porosity of the outer layer on it. This layer is most likely a result of oxidation of the hydrogen-enriched layer of the alloy formed during cathodic charging. 

## 4. Discussion

### 4.1. Physical Model and Basic Equations

The qualitative picture of the oxidation of zirconium alloys in high-temperature electrolytes suggests the following generalized reaction of the protective layer growth:

$$\begin{array}{l} \mathrm{Alloy}/\mathrm{oxide}\;\mathrm{interface}:\;Zr_{m}\overset{k_{1}}{\to }Zr_{Zr}+2V_{O}^{••}+4e^{\prime }\;(\mathrm{metal}\;\mathrm{oxidation})\\ \mathrm{Oxide}/\mathrm{coolant}\;\mathrm{interface}:\;2V_{O}^{••}+2H_{2}O+4e^{\prime }\overset{k_{2}}{\to }2O_{O}+4H_{oxide}\;(\mathrm{reduction}\;\mathrm{of}\;\mathrm{water}\;\mathrm{and}\;\mathrm{entry}\;\mathrm{of}\;\mathrm{oxygen}\\ \mathrm{ions}\;\mathrm{and}\;\mathrm{hydrogen}\;\mathrm{atoms}\;\mathrm{into}\;\mathrm{the}\;\mathrm{oxide}),\; \end{array}$$

where *Zr_m_* is a zirconium atom in the alloy, *Zr_Zr_* and *O_O_* are zirconium and oxygen positions in the crystal lattice of *ZrO*_2_, *V_O_*^••^ is an oxygen vacancy in this lattice, and *k*_1_ and *k*_2_ are the rate constants of the interfacial reactions. The above processes require transport of oxygen and electrons through the oxide. Oxygen is transferred by a vacancy diffusion/migration mechanism along grain boundaries of already formed oxide, while electrons—by a polaron hopping mechanism via secondary phases and/or hetero-valent substituted ions (e.g., Nb^II^_Zr_″, Nb^III^_Zr_′, Nb^V^_Zr_^•^). At the barrier film/coolant interface, restructuring of the barrier film and formation of a secondary outer oxide layer proceeds. This restructuring is assumed to proceed by a dissolution/deposition mechanism, the rate of which depends on the concentration of alkali:

$$ZrO_{2}+OH^{-}\overset{k_{d}}{\to }HZrO_{3}^{-}\overset{}{\to }Zr{\left(OH\right)}_{4}$$

As mentioned above, the flux of oxygen by vacancy mechanism *J_O_*(*x*,*t*) is determined by diffusion and migration:

(1) $$J_{O}(x,t)=-D_{O}\frac{\partial c_{O}(x,t)}{\partial x}-2\frac{FE}{RT}D_{O}c_{O}(x,t)$$

In this equation, *c_O_*(*x*,*t*) is the concentration of oxygen vacancies as a function of time *t* and distance *x* in the oxide, ***E*** is the electric field strength, and *D_O_* the diffusion coefficient of oxygen by a vacancy mechanism; *F*, *R*, and *T* have their usual meanings. The steady-state solution of the above equation subject to the boundary conditions *J_O_*(*L*) = *k*_1_, *J_O_*(0) = *k*_2_*c_O_*(0) in a coordinate system where *x* = 0 is at the oxide/electrolyte interface and *x* = *L_b_* at the alloy/oxide interface, *L_b_* being the barrier oxide thickness, leads to the following expression for the concentration profile of oxygen vacancies:

(2) $$c_{O}(x)=2k_{1}\left[\frac{e^{-2\frac{FE}{RT}x}}{k_{2}}+\frac{RT}{2FED_{O}}\right]$$

The total impedance of the coolant/outer layer/barrier layer/alloy layer/coolant system can be written as

(3) $$Z=R_{el}+Z_{out}+Z_{b}=R_{el}+Z_{out}+{\left(Z_{e}^{-1}+Z_{ion}^{-1}\right)}^{-1}$$

where *R_el_* is the uncompensated electrolyte resistance and *Z_out_* is the impedance function describing the dielectric properties of the outer oxide film. Following previous treatments [19,20], the impedance of the outer layer, *Z_out_*, is described with the so-called Havriliak-Negami impedance [23]

(4) $$Z_{out}=\frac{R_{out}}{{\left[1+{\left(j\omega R_{out}C_{out}\right)}^{u}\right]}^{n}}$$

where $R_{out}$ is the electrical resistance of the outer layer, $C_{out}$—its capacity, and *u* and *n* are constants with values between 0 and 1. Equation (4) describes charge transport through a porous layer by migration along conductive linear defects with no concentration gradient at difference to the diffusion-migration mechanism in the barrier layer described by Equation (1).

In Equation (3), *Z_e_* is the impedance function describing the dielectric properties of the barrier oxide layer on the surface of the zirconium alloy, which is related to the variation of the steady-state concentration of oxygen vacancies, playing the role of donors, with distance in the oxide. Using Equation (2) as a starting point, the following expression is obtained for *Z_e_* within the frames of the MCM [21]

(5) $$Z_{e}=\frac{RT\varepsilon \varepsilon_{0}}{2j\omega FE}\ln\left[\frac{1+j\omega \frac{RT}{F^{2}D_{e}}\frac{k_{2}}{k_{1}}\varepsilon \varepsilon_{0}e^{2\frac{FE}{RT}L}}{1+j\omega \frac{RT}{F^{2}D_{e}}\frac{k_{2}}{k_{1}}\varepsilon \varepsilon_{0}}\right]$$

where *ε* is the dielectric constant of the oxide, assumed to be equal to 22 [20], *ε*_0_ is the dielectric permittivity of vacuum and *D_e_* is the diffusion coefficient of electronic carriers. 

In turn, the impedance of ion transport, *Z_ion_*, is obtained by solving Equation (1) in the frequency domain for a low-amplitude sinusoidal perturbation [21]

(6) $$Z_{ion}^{}=R_{t}+\frac{{\left(RT\right)}^{2}}{4F^{3}ED_{O}c_{O}^{}(L_{b})(1-\alpha )\left[1+\sqrt{1+\frac{j\omega {\left(RT\right)}^{2}}{F^{2}E^{2}D_{O}}}\right]}$$

where R_t_ is charge transfer resistance inversely proportional to the exchange current of the zirconium oxidation reaction, *c_O_*(*L*) is the concentration of oxygen vacancies at the alloy/oxide interface and α the part of the potential consumed at the film/coolant interface compared to film bulk. In the simplest case, it can be assumed that *α* = 0, i.e., the entire potential drop in the alloy/oxide/electrolyte system is located in the barrier layer. On the other hand, based on Equation (2), the concentration *c_O_*(*L*) can be approximated as

(7) $$c_{O}(L_{b})=2k_{1}\left[\frac{e^{-2KL}}{k_{2}}+\frac{RT}{2FED_{O}}\right]\approx \frac{RTk_{1}}{FED_{O}}$$

Finally, inserting the assumed value of α and the approximate expression for $c_{O}(L_{b})$ in Equation (6), an equation for the impedance of ion transport across the oxide barrier sublayer is obtained.

(8) $$Z_{ion}=R_{t}+\frac{RT}{4F^{2}k_{1}\left[1+\sqrt{1+\frac{j\omega {\left(RT\right)}^{2}}{F^{2}E^{2}D_{O}}}\right]}$$

The total impedance transfer function of the system, then, can be expressed by substituting Equations (4), (5) and (8) in Equation (3):

(9) $$Z=R_{el}+\frac{R_{out}}{{\left[1+{\left(j\omega R_{out}C_{out}\right)}^{u}\right]}^{n}}+{\left[{\left(\frac{RT\varepsilon \varepsilon_{0}}{2j\omega FE}\ln\left[\frac{1+j\omega \frac{RT}{F^{2}D_{e}}\frac{k_{2}}{k_{1}}\varepsilon \varepsilon_{0}e^{2\frac{FE}{RT}L}}{1+j\omega \frac{RT}{F^{2}D_{e}}\frac{k_{2}}{k_{1}}\varepsilon \varepsilon_{0}}\right]\right)}^{-1}+{\left(R_{t}+\frac{RT}{4F^{2}k_{1}\left[1+\sqrt{1+\frac{j\omega {\left(RT\right)}^{2}}{F^{2}E^{2}D_{O}}}\right]}\right)}^{-1}\right]}^{-1}$$

### 4.2. Parameter Estimation

**Figure 10:**
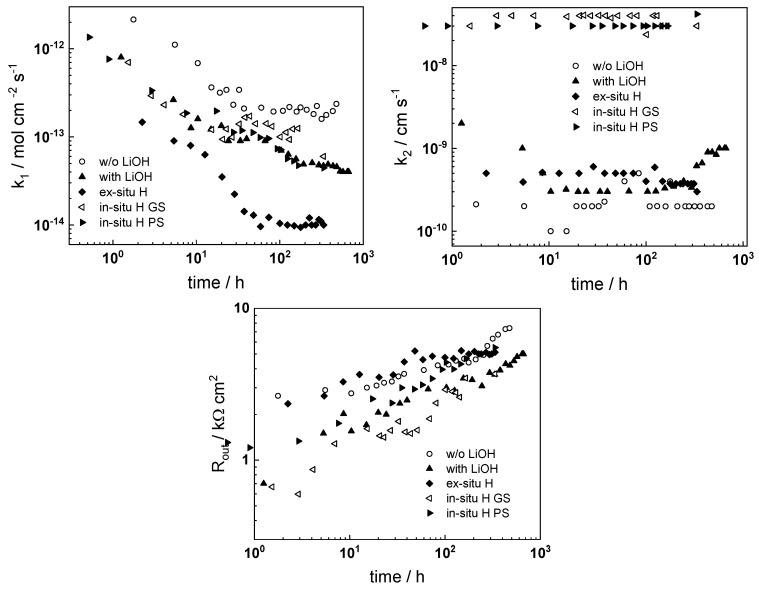
Parameters of interfacial reactions as a function of time and oxidation conditions.

**Figure 11:**
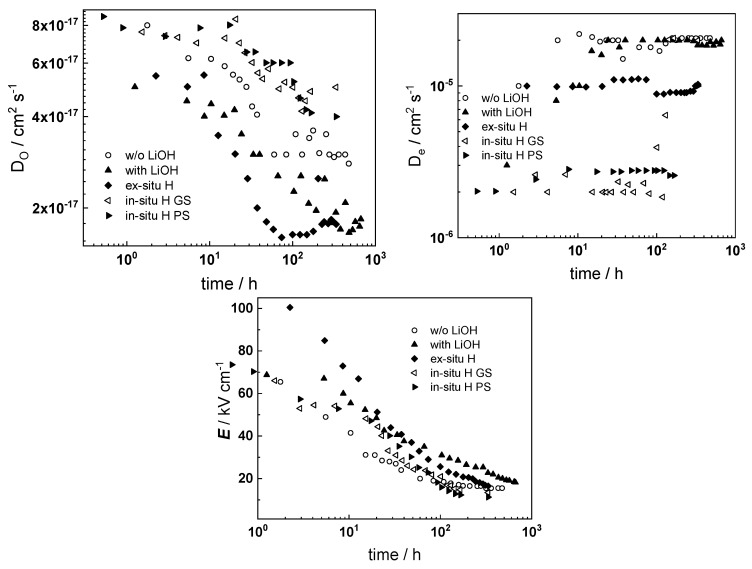
Defect transport parameters as a function of time and oxidation conditions.

The experimental data were fitted to the transfer function expressed by Equations (3)–(8) using the procedure described in the Experimental section. The comparison of experimental and best-fit calculated impedance spectra (Figure 1, Figure 2, Figure 3, Figure 4 and Figure 5) demonstrates the ability of the proposed model to reproduce quantitatively both the magnitude and the frequency distribution of the impedance function. Therefore, estimates of the kinetic parameters can be considered viable and suited to reproduce oxidation rates of the material in a simulated WWER coolant. The dependences of the main parameters of the processes at the alloy/oxide and oxide/coolant interfaces (rate constants *k*_1_ and *k*_2_, and resistance of the outer oxide layer *R_out_* and *C_out_*), as well as those characterizing defect transport (electric field intensity ***E***, diffusion coefficients of oxygen anions and electrons) are presented in Figure 10 and Figure 11 as depending on oxidation time. The estimated values of the metal oxidation rate constant at the alloy/oxide interface, the electric field strength, and the diffusion coefficients in the oxide are in line with our previous work [20].

Based on the dependence of the kinetic and transport parameters with oxidation time, the following conclusions on their relative significance in the overall oxidation process can be drawn:

- The rate constant of metal oxidation at the alloy/oxide interface decreases significantly with time indicating that the corrosion rate decreases with increasing film thickness. Nearly constant values are reached after ca. 100–200 h of oxidation, i.e., a quasi-steady state is achieved. On the other hand, the rate of incorporation of oxygen at the oxide/coolant interface is almost independent on time, suggesting that this process is of secondary significance when compared to metal oxidation at the inner interface;
- The charge transfer resistance at the oxide/coolant interface increases with oxidation time, which means that the respective rate of water reduction decreases. This is in line with the decrease of metal oxidation rate leading to a smaller electron supply rate for the coupled cathodic reaction, taking into account the fact that the diffusion coefficient of electrons does not exhibit any dependence on oxidation time.

A rationalization of the decrease of the oxygen diffusion coefficient and field strength in the oxide with oxidation time (or equivalently, film thickness) is attempted in the next section. 

### 4.3. Influence of Internal Stresses on Oxygen Transport by Vacancy Mechanism

Atomistic simulations by density functional theory (DFT) were reported to estimate the effect of internal stresses on the energy of formation and migration of oxygen vacancies during oxide film growth on zirconium alloys [24]. Table 3 shows the variations in the formation energies of oxygen vacancies in the m-and t-ZrO_2_, as well as migration energies t-ZrO_2_, depending on stress direction with a value of 1 GPa. This value corresponds to the limiting stress in oxides formed on Zircaloy-4 as estimated by synchrotron X-ray diffraction [25]. 

Based on these calculations and the expressions for the diffusion coefficient of oxygen via vacancies in the presence and absence of stress:

(10) $$\begin{array}{l} D_{O,\sigma }=\frac{1}{6}z\nu a^{2}\exp\left(-\frac{E_{f}+\Delta E_{f}}{RT}\right)\exp\left(-\frac{E_{m}+\Delta E_{m}}{RT}\right)\\ D_{O,\sigma =0}=\frac{1}{6}z\nu a^{2}\exp\left(-\frac{E_{f}}{RT}\right)\exp\left(-\frac{E_{m}}{RT}\right) \end{array}$$

ratios of diffusion coefficients in the presence and absence of compressive stress were estimated:

(11) DO,σDO,σ=0=exp−ΔEf+ΔEmRT=exp−ΔERT

The values of this ratio at a stress of 1 GPa are presented in Table 4.

The evolution of compressive stress with oxide thickness (i.e., with oxidation time) was adopted from synchrotron X-ray diffraction data of zirconium alloys oxidized at 350 °C in a simulated primary coolant [25]. In that paper. internal stresses were reported to increase quasi-linearly with thickness and reach limiting values close to 1 GPa, i.e., the corresponding diffusion coefficients for oxides of different thicknesses was corrected using data presented in Table 4 and the corresponding compressive stress-time dependence.

### 4.4. Influence of Space Charge of Substitutional Ions on the Field Strength in the Oxide

According to the scheme of the oxidation process, transport of oxygen proceeds from the outer to the inner interface by a vacancy mechanism, and electrons transfer in the opposite direction (they are consumed by the reaction of reduction of water at the oxide/coolant interface) [5]. Since transport of electrons is significantly faster than that of oxygen ions, a space charge builds up in the oxide and creates an additional electric field, influencing the transport of current carriers. In addition, hetero-valent impurities, such as niobium ions in different oxidation states (from two to four, according to synchrotron X-ray absorption fine structure data [26,27]), that are present in the oxide partly compensate the space charge. The generalized expression for the field strength has the form:

(12) $$E(L)=E_{0}+\frac{e}{\varepsilon \varepsilon_{0}}\int_{0}^{L}\sum_{i}z_{i}c_{i}(x)dx$$

Assuming that hydrogen is transferred to the oxide in atomic form (i.e., has no charge), the sum under the integral is written as

(13) $$\sum_{i}z_{i}c_{i}(x)=2c_{o}(x)-c_{e^{\prime }}(x)-z_{Nb}c_{Nb}(x)$$

Introducing a homogeneous variation of defect concentrations, we obtain a simplified expression:

(14) $$E(L)=E_{0}+L\frac{F\Delta c(0)}{\varepsilon \varepsilon_{0}\left(1+\frac{L}{x_{0}}\right)}$$

in which $x_{0}=\frac{\varepsilon \varepsilon_{0}RT}{{\left(2F\right)}^{2}ac_{o}(0)}$ is the so-called field shielding parameter from space charge [28]. 

### 4.5. Kinetics of Barrier Oxide Growth 

The thickness of the barrier oxide film increases with time according to a logarithmic law derived based on the MCM.

(15) $$L_{b}(t)=L_{b,t=0}+\frac{1}{b}\ln\left[1+\Omega k_{1}^{}be^{-bL_{b,t=0}}t\right],\;b=\frac{2\alpha_{1}FE}{RT}$$

α_1_ being the transfer coefficient of Zr oxidation reaction at the alloy/barrier layer interface, *L_b,t_*_=0_—the initial barrier layer thickness and Ω the molar volume of the barrier oxide. 

### 4.6. Model Validation Based on Computational Results

**Figure 12:**
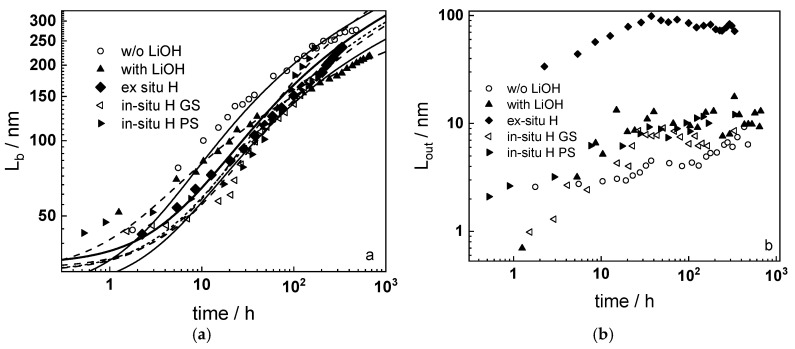
Thicknesses of the barrier oxide layer (**a**) and the outer layer (**b**) estimated from EIS depending on the oxidation time. Lines in (**a**) are best-fit calculations to Equation (15).

Figure 12 shows the dependences of the barrier and outer layer thicknesses on oxidation time under different experimental conditions, evaluated by quantitative interpretation of the impedance spectra. The lines represent a non-linear regression of this type of data to Equation (15), with a very good fit, i.e., the proposed equation adequately describes thickness of the protective layer—time dependences. Thickness estimates obtained from analysis of impedance spectra are in good agreement with average values obtained from interpretation of GDOES depth profiles and cross-sectional microscopic observations. The outer layer thickness is smaller than that of the barrier layer, with the notable exception of the sample that was pre-hydrogenated ex-situ in 0.1 M KOH. A tentative explanation is that in this case, the outer layer is formed at the expense of a hydrogen-rich layer in the underlying alloy. 

**Figure 13:**
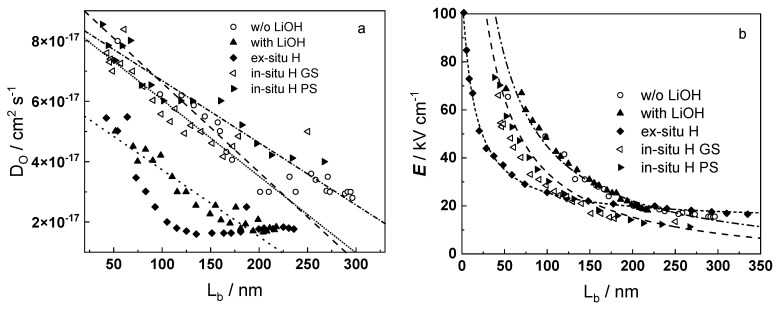
(**a**) Dependence of oxygen diffusion coefficient on oxide thickness under different conditions. Points-calculated values based on nonlinear regression of impedance spectra; lines-regression based on the assumption for linear increase of compressive stresses with oxide layer thickness; (**b**) Dependence of the field strength on the thickness of the oxide obtained under different conditions. Points-calculated values based on nonlinear regression of impedance spectra, lines-nonlinear regression by Equation (14).

Dependences of the oxygen diffusion coefficient on barrier layer thickness in different experimental conditions are presented in Figure 13. The diffusion coefficient decreases linearly with thickness, which is due to a linear increase of compressive stress in the oxide with thickness, causing an increase in the energy of the formation/migration of oxygen vacancies. A significant deviation from the predicted dependence was observed only for the samples pre-hydrogenated for 24 h in 0.1 M KOH, which could be explained by the formation of a hydrogen-rich layer during charging. The oxidation of such a layer can be assumed to proceed by a mechanism that is different from the thickening of the native oxide on untreated samples.

Figure 13b illustrates the thickness dependences of the field strength in the oxides. The solid lines represent the results of non-linear regression of these dependences according to Equation (14). The computational results show agreement with the estimates determined from impedance spectra, which also validates the hypothesis of the proposed approach on the influence of the space charge of mobile point defects and immobile aliovalent impurities on field strength in the growing oxide.

**Figure 14:**
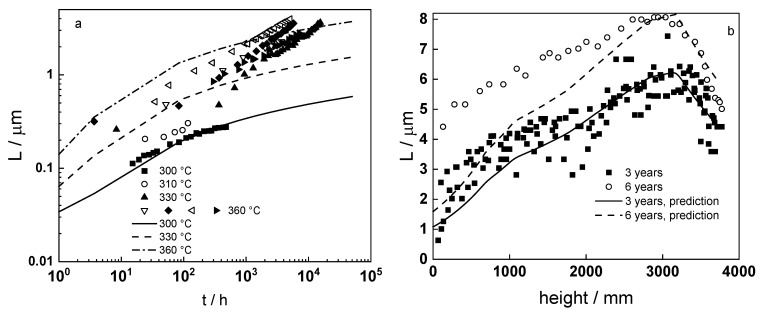
(**a**) Thickness of oxide layers on Zr-1%Nb as depending on temperature and time of oxidation; (**b**) Distribution of oxide thickness on Zr-1%Nb fuel cladding by fuel rod height after 3 and 6 years of service in a WWER-1000 reactor: points-experimental data from various sources, lines-model predictions.

A comparison of the model predictions with oxide thickness vs. time data for Zr-1%Nb alloys in WWER and PWR coolants at different temperatures [18,19,25,27,29,30] is presented in Figure 14a. The quality of prediction is satisfactory, considering that calculations on the basis of parameterization of impedance spectra give a measure of the instantaneous oxide thickness, whereas literature data based on weight gain measurements and microscopic observations estimate the cumulative thickness of the growing layer. Notably, the model predicts the growth of the barrier film, whereas the formation of an external layer with pores and cracks that does not limit further oxidation of the alloy is not quantified.

As a next step of model verification, its predictions are compared with data on oxide thickness distribution by fuel rod height after three and six years of service [31] in a WWER-1000 reactor (Figure 14b), using the appropriate temperature distribution [32]. The quality of prediction is quite good for three years of service and becomes somewhat worse after six years. This could be due to the fact that similar temperature-height distributions were used in both cases. 

In general, it can be concluded that in-situ measurements using EIS and their quantitative interpretation by the MCM represent an important step towards the elucidation of the mechanism of compact protective layer growth on zirconium alloy as fuel cladding and internals in nuclear reactors. The quantification of hydrogen pick-up and hydride formation, as well as the prediction of their effects on service life of zirconium alloys, is underway and will be reported in the near future. 

## 5. Conclusions

In the present paper, oxidation of Zr-1%Nb alloy in simulated primary coolants of WWER reactor is studied with a combination of in-situ electrochemical measurements and ex-situ analytical methods of thin film characterization. A new version of a kinetic model of oxide growth including the effects of compressive stress and ionic space charge on defect transport is proposed to interpret the results obtained. The following main conclusions can be drawn from the experimental results and model calculations:

- Electrochemical impedance measurements allow to discernment of the contribution of barrier and outer layer conductivities, kinetics of interfacial reactions, and ionic defect transport in the overall oxidation process;
- Layer thicknesses estimated from EIS are in good agreement with those determined by ex-situ analysis techniques (GDOES and SEM). The thickness of the barrier protective layer is much larger than that of the outer layer except for oxidation of ex-situ pre-hydrogenated samples;
- The proposed kinetic model is able to reproduce quantitatively the impedance spectra as depending on oxidation time in a variety of experimental conditions;
- Taking into account the influence of compressive stress and space charge in the oxide allows rationalization of the dependences of the diffusion coefficient of oxygen via vacancy mechanism and the field strength in the oxide on film thickness, or equivalent oxidation time. Barrier thickness vs. time data are successfully interpreted with the same set of kinetic parameters furnishing further credibility to the model.

## Figures and Tables

**Table 1:** Chemical composition of the studied material.

Element	Zr	Nb	Sn	Fe	N	C	O
Content/wt.%	Base	1.05	0.04	0.05	<0.01	0.02	0.10

**Table 2:** Comparison of estimates of oxide thickness obtained by GDOES and SEM.

Experiment	Oxide Thickness (GDOES) µm/2 Points	Oxide Thickness (SEM) µm/22 Points
w/o LiOH, 720 h	0.44 ± 0.01	0.40 ± 0.04
with LiOH, 720 h	0.31 ± 0.01	0.27 ± 0.04
w/o LiOH, ex-situ charged, 360 h	0.32 ± 0.01	0.33 ± 0.04

**Table 3:** Energy of oxygen vacancy formation (*E_f_*) and migration (*Ev*) for different ZrO_2_ modifications depending on the direction of a compressive stress of 1 GPa [24].

Oxide Structure	Isotropic Compression	Compression in Direction a	Compression in Direction b	Compression in Direction c
*E_f_* m-ZrO_2_(O1)/eV	0.010	0.012	0.011	0.032
*E_f_* m-ZrO_2_(O2)/eV	0.012	0.016	0.010	0.022
*E_f_* t-ZrO_2_/eV	0.015	0.011		0.006
*E_V_* t-ZrO_2_/eV	0.034	0.070	0.008	0.030

**Table 4:** Ratio of diffusion coefficients of oxygen vacancies in the presence and absence of 1 GPa compressive stresses with different orientation.

Ratio of Diffusion Coefficients	Isotropic Compression	Compression in Direction a	Compression in Direction b	Compression in Direction c
DO,σDO,σ=0	0.37	0.19	0.68	0.48

## Data Availability

The data presented in this study are available on request from the corresponding author.
